# Independent estimation of the frequency of rare CNVs in the UK population confirms their role in schizophrenia

**DOI:** 10.1016/j.schres.2011.11.004

**Published:** 2012-03

**Authors:** Detelina Grozeva, Donald F. Conrad, Chris P. Barnes, Matthew Hurles, Michael J. Owen, Michael C. O'Donovan, Nick Craddock, George Kirov, WTCCC

**Affiliations:** a MRC Centre for Neuropsychiatric Genetics and Genomics, School of Medicine, Cardiff University, Heath Park, Cardiff, CF14 4XN, UK; b The Wellcome Trust Sanger Institute, Wellcome Trust Genome Campus, Hinxton, Cambridge, CB10 1SA, UK

**Keywords:** CNV, Schizophrenia, WTCCC

## Abstract

**Background:**

Several large, rare chromosomal copy number variants (CNVs) have recently been shown to increase risk for schizophrenia and other neuropsychiatric disorders including autism, ADHD, learning difficulties and epilepsy.

**Aims:**

We wanted to examine the frequencies of these schizophrenia-associated variants in a large sample of individuals with non-psychiatric illnesses to better understand the robustness and specificity of the association with schizophrenia.

**Methods:**

We used Affymetrix 500K microarray data from 10,259 individuals from the UK Wellcome Trust Case Control Consortium (WTCCC) who are affected with six non-psychiatric disorders (coronary artery disease, Crohn's disease, hypertension, rheumatoid arthritis, types 1 and 2 diabetes) to establish the frequencies of nine CNV loci strongly implicated in schizophrenia, and compared them with the previous findings.

**Results:**

Deletions at 1q21.1, 3q29, 15q11.2, 15q13.1 and 22q11.2 (VCFS region), and duplications at 16p11.2 were found significantly more often in schizophrenia cases, compared with the WTCCC reference set. Deletions at 17p12 and 17q12, were also more common in schizophrenia cases but not significantly so, while duplications at 16p13.1 were found at nearly the same rate as in previous schizophrenia samples. The frequencies of CNVs in the WTCCC non-psychiatric controls at three of the loci (15q11.2, 16p13.1 and 17p12) were significantly higher than those reported in previous control populations.

**Conclusions:**

The evidence for association with schizophrenia is compelling for six rare CNV loci, while the remaining three require further replication in large studies. Risk at these loci extends to other neurodevelopmental disorders but their involvement in common non-psychiatric disorders should also be investigated.

## 1 Introduction

Schizophrenia has a strong genetic component with heritability estimates of up to 80% and ~ 10-fold elevated recurrence risk in first-degree relatives (Gottesman, 1991; Cardno and Gottesman, 2000). Since 2008, several specific copy number variants (CNVs) that represent deletions or duplications of > 1000 bp of DNA, have been shown to increase susceptibility to schizophrenia as well as other neurodevelopmental disorders (International Schizophrenia Consortium, 2008; Stefansson et al., 2008; Walsh et al., 2008; Xu et al., 2008; Kirov et al., 2009a, 2009b; McCarthy et al., 2009; Sebat et al., 2009; Moreno-De-Luca et al., 2010; Mulle et al., 2010; Levinson et al., 2011). All implicated CNVs are very rare, occurring in about 1:150 to 1:1000 individuals with schizophrenia, frequencies about 2 to 10 times higher than in controls. However, because of their rarity, the frequencies in the general population have not yet been confidently established and estimated effect sizes could change substantially if additional CNVs are found in other control populations. Furthermore, the discovery of most loci depended heavily on the frequencies found in large control samples from the Icelandic population, therefore it is important to evaluate these frequencies in other large control samples, in order to draw definitive conclusions. Here we used data from one of the largest genetic studies, the Wellcome Trust Case Control Consortium (WTCCC), to examine the frequency of these schizophrenia-associated loci in over 10,000 people from the UK population who are affected with non-psychiatric disorders. These subjects have not been used as controls in any previous CNV studies of schizophrenia and so provide independent estimates of the frequencies of the CNVs in subjects without neurodevelopmental disorders. These subjects cannot be strictly described as “controls”. They are not a sample representative of the general population either, as they suffer with a variety of common medical disorders. However, if the CNVs we test increase the risk to develop some of these disorders, this would in fact make our comparison with schizophrenia more conservative. We will refer to these individuals as the “WTCCC reference set”.

## 2 Method

### 2.1 Subjects

We studied data from 10,259 individuals that passed quality control filtering. Subjects were ascertained by the WTCCC with coronary artery disease (CAD, n = 1855), Crohn's disease (CD, n = 1450), hypertension (HT, n = 1864), rheumatoid arthritis (RA, n = 1374), type 1 diabetes (T1D, n = 1903) and type 2 diabetes (T2D, n = 1813). They are of White European ancestry and live in the UK. Extensive information about the samples and method of genotyping has been published previously (Wellcome Trust Case Control Consortium, 2007). The WTCCC study also includes population controls: people born in one week in 1958, and individuals from the National Blood Transfusion Service (NBS). These ~ 3000 control individuals have been used already to establish the associations of several CNV loci with schizophrenia (Kirov et al., 2009a; McCarthy et al., 2009) and are therefore not included in the current analysis. The 1650 bipolar disorder cases from the WTCCC study are also excluded from analysis as there is strong evidence for a degree of genetic overlap between bipolar disorder and schizophrenia (Lichtenstein et al., 2009). The results on CNVs in the bipolar disorder patients have been published elsewhere (Grozeva et al., 2010).

### 2.2 Genotyping, CNV detection and CNV quality control

Genotyping was performed with Affymetrix 500K arrays that cover the genome with ~ 500,000 single nucleotide polymorphisms (SNPs), as described previously (Wellcome Trust Case Control Consortium, 2007). The signal intensities at each SNP are used to infer the copy number state. Deletions and duplications result respectively in lower and higher signal intensities at SNPs in the affected regions. The pre-CNV calling normalisation procedure and the CNV-calling methods have been described elsewhere (Olshen et al., 2004; Price et al., 2005; Barnes et al., 2008). As is now routine for this type of analysis, several quality control (QC) filtering steps were applied. These were removal of samples in which the raw data were of such poor quality that they were rejected for SNP analyses, or in which the intensity data was highly variable leading to excessive CNV calling (Kirov et al., 2009a). One potential bias in copy number analysis could arise when comparing samples derived from cell lines and blood as the process of generating cell lines can result in structural rearrangements (Wang et al., 2007). To minimise this effect, additional QC measures were undertaken: removing very large events (> 5 Mb), applying a “wave correction” during normalisation, and removing CNVs from whole chromosomes where the median chromosomal intensity was an outlier from the distribution, as it was observed that such events were enriched among DNA from cell-lines, suggesting that these are cell-line artefacts (Wang et al., 2007). CNV calling and QC filtering measures were performed at the Wellcome Trust Sanger Institute and subsequently the data were made publicly available.

Previously we performed similar CNV analyses on other phenotypes from the WTCCC dataset (schizophrenia, n = 471, bipolar disorder, n = 1697, and controls, n = 2806) (Kirov et al., 2009a; Grozeva et al., 2010). All these sample sets were genotyped in the same pipeline along with CAD, CD, RA, HT, T1D and T2D (Wellcome Trust Case Control Consortium, 2007; O'Donovan et al., 2008a). This work allowed us to compare the CNV calling performed in house (Kirov et al., 2009a; Grozeva et al., 2010), with that performed independently by the WTCCC. Agreement between the calls for these large CNVs was 100% (35 schizophrenia-associated CNVs called by both teams). Thus we can conclude that we are not introducing bias by analysing CNVs called at different sites by different analytical methods. This conclusion is in agreement with the widely accepted view that CNVs larger than 500,000 bp are reliably called with any high-density microarrays. All CNVs discussed in this report are > 600,000 bp long and covered with between 25 to several hundred probes on the arrays, allowing a reliable comparison with the previous studies.

All chromosomal positions presented in this paper are according to NCBI Build 36 (hg18), March 2006 of the UCSC Genome Browser.

### 2.3 Analysed loci

We chose to analyse loci that have received replicated evidence from multiple large studies (International Schizophrenia Consortium, 2008; Stefansson et al., 2008; Kirov et al., 2009a, 2009b; McCarthy et al., 2009; Moreno-De-Luca et al., 2010; Mulle et al., 2010; Ingason et al., 2011; Levinson et al., 2011). The vast majority of samples were of white European ancestry, with the exception of 1274 schizophrenia cases and 963 controls of African American origin who were included in the study of Levinson and colleagues (Levinson et al., 2011). Details of the previous evidence for each locus are presented in the Results and Discussion sections and Table 1. We compared the frequency of CNVs in the WTCCC reference set with that in the schizophrenia cases taken from the largest published studies or reviews (for 1q21.1, 15q13.3, 16p11.2, 17p12, 17q12 and 22q11.2) or from combined data from studies that had at least 1000 cases and 1000 controls (for 3q29, 15q11.2 and 16p13.1), excluding any individuals who had taken part in more than one of these studies (International Schizophrenia Consortium, 2008; Kirov et al., 2009a; McCarthy et al., 2009; Moreno-De-Luca et al., 2010; Mulle et al., 2010; Ingason et al., 2011; Levinson et al., 2011). Deletions at the *NRXN1* gene, also implicated as a risk factor for schizophrenia, (Kirov et al., 2009b) or at other individual genes implicated recently (Levinson et al., 2011) were not analysed as the CNVs affecting them are usually much smaller than 500,000 bp and can be missed with the lower-resolution array used in the current study. We did not attempt a formal meta-analysis, which would not be appropriate, as the new set of studied individuals does not include people affected with schizophrenia. Therefore, for uniformity, we present all results with a pooled analysis of all available individuals performed with the Fisher's Exact Test. Formal meta-analyses results are available for some of the published loci and the odds ratios (OR) and p-values reported by these two statistical methods in the previous studies are very similar (Levinson et al., 2011).

## 3 Results

Results are presented in Table 1. We present the frequency of the CNVs in schizophrenia cases and controls (columns 5 and 2) derived from the appropriate reviews (cited in the last column). Where a review of the majority of the data was not available (in the case of 3q29, 15q11.2 and 16p13.1), we combined the results from the largest published reports (listed in the last column), after excluding samples used in more than one study, where appropriate. The studies in Table 1 comprise the bulk of available high-quality CNV data, with > 5000 individuals with schizophrenia and > 37,000 controls for each locus. Despite the smaller size of the WTCCC reference set, compared to the size of the previous control populations, six out of the nine CNVs received statistically significant support for association with schizophrenia. Details on each locus are presented in the Discussion. The frequencies of three CNVs were significantly higher than those reported in previous control populations (15q11.2, 16p13.1 and 17p12, p-values presented in column 4 of Table 1), most notably for the 16p13.1 duplication, p = 0.001, which is also the only p-value that would survive a Bonferroni correction for multiple testing of nine independent events (p = 0.006).

Table 2 presents the numbers of the observed CNVs separately for each disease cohort comprising the WTCCC reference set. There are no statistically significant differences in the frequencies of the CNVs between any cohorts, but this is expected, given the small sample size of each cohort and the rarity of the CNVs.

## 4 Discussion

Several large CNVs have previously received strong replicated support for association with schizophrenia: deletions at 1q21.1 (International Schizophrenia Consortium, 2008; Stefansson et al., 2008), 2p16.3, affecting the *NRXN1* gene (Kirov et al., 2008, 2009b; Rujescu et al., 2009), 3q29 (Mulle et al., 2010; Levinson et al., 2011), 15q11.2 (Stefansson et al., 2008; Kirov et al., 2009a), 15q13.3 (International Schizophrenia Consortium, 2008; Stefansson et al., 2008), 17p12 (Kirov et al., 2009a), 17q12 (Moreno-De-Luca et al., 2010) , 22q11.2 (the Velo-Cardio-Facial Syndrome (VCFS) region) (Bassett and Chow, 2008; International Schizophrenia Consortium, 2008; Stefansson et al., 2008; Levinson et al., 2011) and duplications at 16p11.2 (McCarthy et al., 2009) and 16p13.1 (Ingason et al., 2011). These studies used a total of between ~ 5000–11,000 cases and ~ 37,000–50,000 controls, and produced strong statistical support for association with disease, with p-values of between 4.3 × 10^− 4^ and 3.8 × 10^− 25^. The CNVs have very low frequencies in controls, and further studies are required both to confirm several of these associations and to establish more accurately their frequencies in patients and in the population of people without neuropsychiatric illness, thus enabling more accurate estimates of effect sizes of risk. Although the numbers of controls were much larger than the ones used in the current study, almost all previous findings were heavily supported by data on > 30,000 controls from a single country (Iceland), therefore a comparison with another large sample of controls from another country appeared to be justified.

Here we studied a sample of 10,259 individuals from the UK, affected with several common non-neuropsychiatric disorders. This dataset allowed us to assess the frequency of these CNVs in an independent sample of comparison subjects. These individuals have not been screened for the presence of schizophrenia or other CNV-related neurodevelopmental disorders. Nevertheless, the prevalence of schizophrenia is only about 1% in the general population, and therefore the use of unscreened reference samples is not expected to make an appreciable difference to the results. Even if 100 of the ~ 10,000 controls have schizophrenia (~ 1%), these will not contribute more than two or three CNVs in total from those listed in Table 1, as their combined frequency in cases is ~ 2%. Furthermore, the presence of people with psychiatric disorders in this reference set would only reduce the strength of association between these CNVs and schizophrenia, therefore the current study provides a conservative estimate of this association. Another ~ 3000 control subjects that are part of the WTCCC study have been used in the identification of several of these loci, and are therefore not included in the current analysis (Kirov et al., 2009a; McCarthy et al., 2009). The combined burden of the examined loci in Table 1 in these ~ 3000 controls does not significantly differ from that in the 10,259 non-psychiatric reference group (Yates corrected χ^2^ p-value = 0.92).

Overall six of the loci remain significantly more common in the subjects affected with schizophrenia, compared to the WTCCC reference set (1q21.1, 3q29, 15q11.2, 15q13.1, 16p11.2 and 22q11.2). This is despite the fact that the new comparison sample is ~ 4 times smaller than the previously used combined control samples. This provides strong additional support that these CNVs are true risk factors for schizophrenia. For the remaining three loci (16p13.1, 17p12 and 17q12) the differences were not statistically significant (Table 1, column 7). When we compared the rate of the studied CNVs in the non-psychiatric WTCCC reference set with the rate in the previous controls (Table 1, column 4) we observed statistically significant higher frequencies in the WTCCC reference set for the following loci: 15q11.2, 16p13.1, and 17p12. This is most notable for the 16p13.1 duplications (Results), with a frequency of 0.24% among the WTCCC reference set, almost an identical frequency to that reported for schizophrenia in previous studies (0.28%). This suggests the possibility the original finding might have been a false-positive.

The difference between the control/ reference sets, and the observations that the rates of CNVs at 16p13.1, 17p12 and 17q12 were not statistically different between schizophrenia cases and the non-psychiatric WTCCC reference set suggest that either these CNVs also increase the risk to develop some of the disorders that comprise the WTCCC sample, or that the odds ratios in the original studies were inflated, or even false-positives, or that the use of the smaller set of controls here is underpowered. In Table 2 we present the numbers of the pathogenic CNVs observed in each disease cohort that comprise the WTCCC reference set in order to examine if these CNVs also increase the risk to develop some of the non-psychiatric disorders. Type 2 diabetes cases had the highest rate of schizophrenia-related CNVs, a cumulative rate of 1.2%. This is intriguing given strong epidemiological evidence for an increased rate of several chronic somatic disorders among patients with schizophrenia, including Diabetes 1 and 2 types, cerebrovascular disorders and myocardial infarction. The cumulative rate of these CNVs in the rest of the cohorts ranged from 0.58 to 0.8. It is of note that the lowest cumulative CNV burden is found for rheumatoid arthritis (0.58%), as there is evidence of inverse co-morbidity between schizophrenia and connective tissue disorders, most notably rheumatoid arthritis (Mors et al., 1999; Laursen et al., 2011). This raises the intriguing question as to whether CNVs are partially responsible for this inverse comorbidity; answering this question will require larger studies of these CNVs in rheumatoid arthritis.

The decrease of the estimated odds ratios for several loci in the new analysis could be also due to the bias known as the “winner's curse” (Zollner and Pritchard, 2007). This arises because if it is underpowered, the first study reporting an association tends to be performed on a sample where the odds ratio happens to be inflated, as explained elsewhere (Craddock et al., 2008). Nevertheless, the comparisons of the frequencies of these CNVs in cases and in the overall ‘control’ populations (those previously used + the new WTCCC reference set) remain highly significant for each locus, with only minor fluctuation of the significant levels of up to one order of magnitude (Table 3).

We will now discuss how the current results relate to previous findings for each locus.

### 4.1 1q21.1 deletion

This 1.4 Mb deletion contains 11 genes and was found to be associated with schizophrenia in the three largest CNVs surveys (International Schizophrenia Consortium, 2008; Stefansson et al., 2008; Levinson et al., 2011). It has also been implicated in developmental delay, dysmorphic features, cardiac abnormalities, and learning disability (Christiansen et al., 2004; Brunetti-Pierri et al., 2008; Mefford et al., 2008; O'Donovan et al., 2008b). Our results confirm their extreme rarity in people who are not affected with neuropsychiatric disorders and give further support for the association with schizophrenia (p = 3.2 × 10^− 5^).

### 4.2 3q29 deletion

Deletions of 3q29, spanning 1.4 Mb and 21 genes, were first reported to be associated with schizophrenia by Mulle et al. (2010). Like many of the other schizophrenia associated CNVs, this variant has been also found to be associated with intellectual disability and autism (Ballif et al., 2008; Mulle et al., 2010). No deletions were observed in the WTCCC reference set, providing further support that this is a risk factor for susceptibility to schizophrenia (p = 0.001).

### 4.3 15q11.2 deletion

The first report of an association between the 0.6 Mb deletions at this locus that span four genes, and schizophrenia was made by Stefansson et al. (2008). The data in Table 1 for this locus were combined from the Kirov et al. review and the study by Levinson et al. (Kirov et al., 2009a; Levinson et al., 2011). The rate of this deletion in the new WTCCC subjects is 0.39%, a higher rate than in the previous controls (0.24%), but lower than among cases (0.57%), thus providing independent support for the association (p = 0.05). Other studies give further support for the role of this CNV in neuropsychiatric disorders (Doornbos et al., 2009; Mefford et al., 2009), with the highest rate of this deletion (1%) found in patients with idiopathic generalised epilepsy (de Kovel et al., 2010). The prevalence and phenotypic manifestations of this CNV need further investigation.

### 4.4 15q13.3 deletion

The association with schizophrenia of this 1.5 Mb deletion that spans seven genes, is supported by the three largest studies in schizophrenia (The Molecular Genetics of Schizophrenia (Levinson et al., 2011), the International Schizophrenia Consortium (International Schizophrenia Consortium, 2008) and Stefansson et al. (2008)) as reviewed by Levinson et al. (2011). The rate of the 15q13.3 deletion in the new WTCCC reference set is very low at 1:2500, confirming the role of this CNV in schizophrenia (p = 0.001). Similar to other loci implicated in schizophrenia, this deletion is associated with other neuropsychiatric phenotypes. Sharp and colleagues described a syndrome presenting with mental retardation, seizures and variable facial dysmorphic features (Sharp et al., 2008). The deletion is found at ~ 1% among patients with idiopathic generalised epilepsy (Dibbens et al., 2009; Helbig et al., 2009) and at 0.17% among individuals suffering with mental retardation, developmental delay and autism (Miller et al., 2009).

### 4.5 16p11.2 duplication

A large meta-analysis observed this 0.6 Mb duplication containing 26 genes at a rate of ~ 1:300 schizophrenia patients and ~ 1:3500 controls (McCarthy et al., 2009). The rate in the new WTCCC reference set (~ 1:2500) is very similar to that among previous controls, and provides strong support for the association with schizophrenia (p = 3.9 × 10^− 6^). The same duplication is also a risk factor for autism, while the reciprocal deletion of this locus is associated with autism and developmental delay (McCarthy et al., 2009). The reciprocal deletion is recognised as one of the most common molecular risk factor for autism, but it does not appear to be a risk factor for schizophrenia (McCarthy et al., 2009). In the current study, four WTCCC reference subjects carried the deletion.

### 4.6 16p13.1 duplication

This 1.2 Mb duplication spanning eight genes was first implicated as a risk factor for autism (Ullmann et al., 2007) and mental retardation (Mefford et al., 2009). Ingason et al. observed a 3-fold excess of duplications and deletions in cases with schizophrenia compared to controls (Ingason et al., 2011). Table 1 combines their results with those from the International Schizophrenia Consortium (2008), resulting in a 3-fold increase in cases: ~ 1:350 patients carry the duplication, vs. ~ 1:1000 controls. In the current study we found a high rate of this duplication in control subjects (~ 1:400), which is quite close to that observed in schizophrenia cases (~ 1:350). As noted above, this at least raises the question whether the first observation was a false-positive result. The carriers in the present study did not come predominantly from a single phenotype that can explain the increased rate (Table 2). It is therefore not clear why there is such a strong difference between the rate in the WTCCC reference set and that in previous controls (0.24% vs. 0.1%, Fisher's exact test p = 0.001). One possibility is that the lower rate in the previous findings was due to exclusion of subjects that had known neuropsychiatric disorders in the study by Ingason et al. (2011), as further studies also support the pathogenic role of this CNV in such disorders. Thus, Mefford et al. detected duplications in 1.1% of individuals with unexplained intellectual disability (Mefford et al., 2009), de Kovel et al. found deletions at this locus in 6/1234 epilepsy patients (0.5%) and in 2/3022 controls (0.07%), OR = 7.4 (de Kovel et al., 2010), and Williams et al. found an excess of duplications (0.84%) in attention-deficit hyperactivity disorder cases (Williams et al., 2010). Perhaps even more intriguing is the strong association of this CNV with aortic dissections (Kuang et al., 2011). The duplication is under strong selection pressure, indicating that it is pathogenic (Rees et al., 2011). Further studies on cases, controls and other phenotypes are needed to resolve the role of this CNV that appears to be pathogenic in general, but not clearly associated with schizophrenia.

### 4.7 17p12 deletion

This 1.4 Mb deletion causes the neurological disorder hereditary neuropathy with pressure palsies (HNPP, MIM #162500) (Chance et al., 1993), while the reciprocal duplication causes Charcot–Marie–Tooth disease Type 1A (CMT1A, MIM #118220) (Lupski et al., 1991). The deletion was found to be 10 times more common in schizophrenia cases than in controls (0.16% vs. 0.015%, p = 5 × 10^− 5^) (Kirov et al., 2009a). These results are based on a smaller sample size than any of the other loci in Table 1. The current study finds a relatively high rate of this deletion in the WTCCC reference set: 7 out of 10,259 individuals, a 4-fold higher rate than in previous controls (p = 0.02). This is still much lower than the rate in schizophrenia patients, however the new findings are not statistically significant (p = 0.11) and weaken the evidence from the previous association. The association still remains highly significant in the overall analysis (Table 3).

### 4.8 17q12 deletion

Deletions of 1.5 Mb at this region have been associated with schizophrenia (Moreno-De-Luca et al., 2010), as well as with autism/neurocognitive impairment (Loirat et al., 2010; Moreno-De-Luca et al., 2010). One of the 15 genes within the locus, *HNF1B*, has been implicated in the renal cysts and diabetes (RCAD) syndrome (Bingham and Hattersley, 2004). This CNV can present also with various medical conditions (macrocephaly, characteristic facial features, genitourinary tract anomalies, recurrent infections and diabetes) (Moreno-De-Luca et al., 2010; Nagamani et al., 2010). Interestingly one of the two WTCCC subjects harbouring this deletion is from the type 2 diabetes cohort. The current results weaken the evidence for this CNV as a susceptibility factor for schizophrenia, although it remains significant in the combined analysis (Table 3) and should be tested in further studies.

### 4.9 22q11.2 deletion

Deletions of 2.4 Mb at this locus constitute a known genomic disorder, 22q11.2 Deletion Syndrome, also known as Velo-Cardio-Facial Syndrome (VCFS) or DiGeorge Syndrome (Shprintzen et al., 1978). This was the first CNV shown to increase the risk for developing psychosis (Murphy et al., 1999). Levinson et al. reported a frequency of ~ 0.32% among 11,400 schizophrenia cases, while no control with the full deletion was found in any control samples (Table 1) (Levinson et al., 2011). No deletions were observed in the WTCCC reference set either, confirming once again the status of this locus as a highly penetrant risk factor for schizophrenia (lower confidence interval for the OR = 44.2, p = 1.1 × 10^− 27^) and other neurodevelopmental disorders.

The current study provides further support for the involvement of deletions at 1q21.1, 3q29, 15q11.2, 15q13.3, 22q11.2 and duplications at 16p11.2 as risk factors for schizophrenia. The evidence implicating deletions at 17p12 and 17q12 and especially duplications at 16p13.1 is weaker and needs further study. All of these loci have also been shown to increase risk for other neuropsychiatric disorders, indicating that these rare, large DNA variants are not phenotype-specific. Furthermore, most carriers of these CNVs do not develop schizophrenia (i.e. they have a limited penetrance for this disorder of 2–7.4%), with the exception of the deletion at 22q11.2 that has an estimated penetrance of 55% (Vassos et al., 2010).

An important limitation of our study is that most of these CNVs cause other medical disorders and therefore could be found among cohorts recruited for other conditions. This could explain why the rate in the WTCCC comparison subjects was higher than in previously analysed controls for several of these loci. While the overall burden of these CNVs appears to be the same in the WTCCC non-psychiatric reference set and ~ 3000 controls (see above), this study has no statistical power to estimate if any individual non-psychiatric disorder in the WTCCC has an increased burden of any single CNV locus discussed here. Judging from the experience in schizophrenia, such associations have to be tested on > 5000 cases from each phenotype.

## Role of funding source

Funding for the project was provided by the Wellcome Trust under awards 076113 and 085475.

## Contributors

Dr. Grozeva performed quality control of the data, analysed the data and wrote the first draft of the manuscript. The Wellcome Trust Case Control Consortium compiled the dataset (Dr. Hurles and Prof. Craddock are principal WTCCC investigators). Drs Conrad, Barnes and Hurles generated the CNV calls using the SNP Affymetrix intensity data and compiled the list of filtered CNV calls. Dr. Kirov initiated the current analysis. Profs Craddock, O'Donovan and Owen contributed to the planning of the study and the writing of the manuscript. All authors edited and approved the final version of the manuscript.

## Conflict of interest

None.

## Figures and Tables

**Table 1:** Loci implicated in schizophrenia.

CNV (position, Mb)^a^	Rate in previous controls^c^	Rate in WTCCC reference set^c^	p-value b: WTCCC vs. previous controls	Rate in SZ cases^c^	p-value b: SZ vs. previous controls OR (95%CI)	p-value b: SZ vs. WTCCC reference set OR (95%CI)	Publication
1q21.1 del	10/47, 321	1/10, 259	0.78	20/11,392	2.2 × 10^− 8^	3.2 × 10^− 5^	(Levinson et al., 2011)
(144.9–146.3)	(0.021%)	(0.01%)		(0.18%)	8.3 (3.7–19.9)	18.8 (2.89–749.3)	
3q29 del	1/40,941	0/10,259	1	9/9323	2 × 10^− 6^	0.001	(Mulle et al., 2010; Levinson et al., 2011)
(197.4–198.8)	(0.002%)	(0%)		(0.097%)	39.6 (5.5–1734)	(2.2–∞)^d^	
15q11.2 del	120/50,108	40/10,259	0.01	68/11,863	4.13 × 10^− 8^	0.05	(Kirov et al., 2009a; Levinson et al., 2011)
(20.2–20.8)	(0.24%)	(0.39%)		(0.57%)	2.4 (1.8–3.3)	1.5 (1–2.2)	
15q13.3 del	9/45,922	4/10,259	0.4	21/10,887	2.0 × 10^− 9^	0.001	(Levinson et al., 2011)
(28.7–30.3)	(0.02%)	(0.039%)		(0.19%)	9.9 (4.3–24.4)	5.0 (1.7–19.9)	
16p11.2 dup	8/28, 406	4/10,259	0.8	26/8590	7.63 × 10^− 11^	3.9 × 10^− 6^	(McCarthy et al., 2009)
(29.5–30.1)	(0.028%)	(0.039%)		(0.3%)	10.8 (4.7–27.6)	7.8 (2.7–30.7)	
16p13.1 dup	38/37,595	25/10,259	0.001	20/7075	4.3 × 10^− 4^	0.65	(ISC, 2008; Ingason et al., 2011)
(15.0–16.2)	(0.10%)	(0.24%)		(0.28%)	2.8 (1.5–4.9)	1.2 (0.6–2.2)	
17p12 del	6/38,884	7/10,259	0.02	8/5089	5 × 10^− 5^	0.11	(Kirov et al., 2009a)
(14.0–15.4)	(0.015%)	(0.068%)		(0.16%)	10.2 (3.1–35.7)	2.3 (0.7–7.5)	
17q12 del	0/47,929	2/10,259	0.06	4/6340	2 × 10^− 4^	0.2	(Moreno-De-Luca et al., 2010)
(31.8–33.3)	(0%)	(0.02%)		(0.06%)	(4.9–∞)^d^	3.2 (0.7–35.8)	
22q11.2 del	0/45,361	0/10,259	1	35/11,400	3.8 × 10^− 25^	3.4 × 10^− 10^	(Levinson et al., 2011)
(17.4–19.8)	(0%)	(0%)		(0.31%)	(35.9–∞)^d^	(8.1–∞)^d^	

a The coordinates (cytogenetic band and position in Mb on the chromosomes according to Build 36) are given in column 1 and exclude flanking low copy repeats.

b All p-values are from Fisher's exact test. Details for each locus are presented in the Discussion. WTCCC reference set denotes the 10,259 individuals with non-psychiatric disorders; SZ: schizophrenia.

c The rates in each population (columns 2, 3 and 5) are presented as numbers of CNV carriers/numbers of all tested individuals.

d The OR for these CNVs cannot be estimated, as no deletion carriers were found in the control populations, ORs are presented as (lower 95%CI-∞).

**Table 2:** Number of CNVs for the studied loci in the separate cohorts comprising the WTCCC reference set.

CNV	RA (1374)	CD (1450)	T1D (1903)	T2D (1813)	CAD(1855)	HT(1864)
1q21.1 del	0	0	0	0	1	0
3q29 del	0	0	0	0	0	0
15q11.2 del	5	3	8	12	6	6
15q13.3 del	0	0	0	2	2	0
16p11.2 dup	0	0	2	0	1	1
16p13.1 dup	2	6	4	5	4	4
17p12 del	1	1	1	2	1	1
17q12 del	0	1	0	1	0	0
22q11.2 del	0	0	0	0	0	0

The numbers of studied individuals are provided in brackets. None of the subjects carried more than one of these CNVs.

**Table 3:** Rate of CNVs in previous studies on schizophrenia and in all control samples (combined previous controls + WTCCC reference set).

CNV (position, Mb)	Rate in SZ cases	Rate in combined controls	p-value: SZ vs. combined controls OR (95%CI)
1q21.1 del	20/11,392	11/57,580	2.9 × 10^− 9^
(144.9–146.3)	(0.18%)	(0.02%)	9.2 (4.2–21.3)
3q29 del	9/9323	1/51,200	4.2 × 10^− 7^
(197.4–198.8)	(0.097%)	(0.002%)	49.5 (6.9–2168)
15q11.2 del	68/11,863	160/60,367	5.7 × 10^− 7^
(20.2–20.8)	(0.57%)	(0.27%)	2.2 (1.6–2.9)
15q13.3 del	21/10,887	13/56,181	2.7 × 10^− 9^
(28.7–30.3)	(0.19%)	(0.023%)	8.3 (4–18.2)
16p11.2 dup	26/8590	12/38,665	1.8 × 10^− 11^
(29.5–30.1)	(0.3%)	(0.031%)	9.8 (4.8–21.3)
16p13.1 dup	20/7075	63/47,854	0.005
(15.0–16.2)	(0.28%)	(0.13%)	2.1 (1.2–3.6)
17p12 del	8/5089	13/49,143	0.0004
(14.0–15.4)	(0.16%)	(0.026%)	5.9 (2.1–15.5)
17q12 del	4/6340	2/58,188	0.001
(31.8–33.3)	(0.06%)	(0.003%)	18.4 (2.6–203)
22q11.2 del	35/11,400	0/55,620	1.1 × 10^− 27^
(17.4–19.8)	(0.31%)	(0%)	(44.2–∞)^a^

a The OR for the 22q11.2 deletion cannot be estimated, as no deletion carriers were found in controls, but the lower confidence interval of the OR is 44.2.
